# Association between visceral/subcutaneous adipose tissue ratio and plasma inflammatory markers and score for cardiovascular risk prediction in a Brazilian cohort: Pró-Saúde Study

**DOI:** 10.1590/1414-431X2021e11521

**Published:** 2021-10-29

**Authors:** W.F. Pereira-Manfro, G.R. de Lima, J.F. Nogueira, M.R.C. Portugal, L.G. Milagres, F.F. Bezerra, E. Faerstein, J.C. Koury

**Affiliations:** 1 Faculdade de Ciências Médicas, Universidade do Estado do Rio de Janeiro, Rio de Janeiro, RJ, Brasil; 2 Instituto de Nutrição, Universidade do Estado do Rio de Janeiro, Rio de Janeiro, RJ, Brasil; 3 Instituto de Medicina Social, Universidade do Estado do Rio de Janeiro, Rio de Janeiro, RJ, Brasil; 4 Departamento de Nutrição, Centro Universitário de Volta Redonda - UniFOA, Volta Redonda, RJ, Brasil

**Keywords:** Adipokines, Algorithmic, Trunk fat mass, Visceral adipose tissue, Subcutaneous adipose tissue

## Abstract

Visceral adipose tissue (VAT) is associated with various metabolic disorders, and adipokines, secreted by adipose tissue, are involved in their pathogenesis. This study investigated associations between VAT/subcutaneous adipose tissue (SAT) ratio, inflammatory markers, and cardiovascular (CV) risk-score in adults. Plasma levels of adipokines, plasma lipid profile, blood pressure, and body composition (using dual-emission x-ray absorptiometry) were determined. CV risk-score based on the American College of Cardiology and the American Heart Association (ACC/AHA) score was calculated in a sample of 309 Brazilian civil servants aged <60 years. Participants' VAT/SAT ratio were categorized into quartiles. Among males, plasma leptin (2.8 ng/mL) and C reactive protein (CRP) (0.2 mg/dL) (P<0.05) levels were higher at P75 and P50 than P5, and the highest calculated CV risk-score was observed at P75 (7.1%). Among females, higher plasma adiponectin levels were observed at P25 (54.3 ng/mL) compared with P75 (36 ng/mL) (P<0.05). Higher plasma CRP levels were observed at P75 (0.4 mg/dL) compared with P5 (0.1 mg/dL) (P<0.05). Higher CV risk-score was observed at P75 (2.0%) compared with P5 (0.7%). In both sexes, VAT and VAT/SAT ratio were directly associated with plasma leptin, CRP, and CV risk-score, and inversely associated with adiponectin; SAT was directly associated with plasma leptin and CRP (P<0.01); interleukin (IL)-10 and CRP were directly associated with adiponectin and leptin, respectively (P<0.05). Among men only, IL-10 (inversely) and CRP (directly) were associated with CV risk-score (P=0.02). Our results strengthened the relevance of the VAT/SAT ratio in cardiovascular risk.

## Introduction

It is well recognized that adipose tissue secretes several bioactive molecules, named adipokines, with different metabolic functions (1). Adipose tissue can be classified according to its distribution in visceral adipose tissue (VAT) and subcutaneous adipose tissue (SAT). VAT is biologically more active, being responsible for adipokines secretion (2,3), and is usually related to type 2 diabetes, dyslipidemia, hypertension, inflammation, and cardiovascular (CV) risk (3-5). Adipokines may act as a pro-inflammatory factor such as leptin and interleukin (IL)-6, which are involved in the pathogenesis of obesity, or as an anti-inflammatory molecule such as adiponectin, which is protective (6).

Body mass index (BMI) has been extensively used to classify obesity (7). However, it is known that abdominal obesity, rather than obesity simply defined by BMI, is more relevant considering CV risk and predisposition for metabolic diseases (8). Nevertheless, the most used algorithms to estimate CV risk-score include BMI, race, sex, blood pressure, lipid profile, smoking status, and ethnicity without information on central obesity (9,10). In order to identify risk for CV diseases, and reduce morbidity and mortality, risk prediction algorithms are used (11). The Reynolds Risk Score includes high-sensitive C-reactive protein (hsCRP) and family history of a heart attack before 60 years of age as risk markers (12,13). However, the most cited sex-specific algorithms are those from the American College of Cardiology/American Heart Association (ACC/AHA) and the General CV disease (CVD) risk developed by Framingham investigators, which have been used to estimate the risk of the first CV event in 10 years (9,10).

Risk factors such as diabetes, hyperlipidemia, and hypertension are well defined in algorithms to calculate the risk of cardiovascular disease. However, some individuals can be affected by a coronary event even in the absence of classic risk factors (14). To our knowledge, no study has examined the relationship among VAT/SAT ratio, inflammatory markers, and CV risk-score in adults. Therefore, the objective of this study was to identify the associations among VAT/SAT ratio with CV risk-score and inflammatory markers in adults regardless of body mass.

## Material and Methods

### Study design and participants

This cross-sectional study was approved by the Ethics in Research Committee of the Institute of Social Medicine at the State University of Rio de Janeiro (CAAE 0041.0.259.000-11). All participants signed a written consent form.

This study was nested within the Pro-Saúde Study, a prospective cohort study of university civil servants in Rio de Janeiro, Brazil, focusing on the investigation of social determinants of health and health-related behavior; details on study population and design were previously described (15). Briefly, four waves of data collection have been conducted among 3,253 participants (1999, 2001-2002, 2006-2007, and 2012-2013). In parallel with wave 4, a subset of 520 participants of wave 1 (baseline), randomly selected within strata of sex, age (less than 50 years *vs* 50 years or more), and educational level (less than high school *vs* high school or more), was invited to perform additional interviews and measurements. Eighty-two participants with metabolic syndrome, 55 participants under immunosuppressive treatment, 68 individuals older than 60 years, and six with low weight (BMI ≤18 kg/m^2^) were excluded from the current study to avoid bias. Finally, a total of 309 participants were enrolled.

### Cardiovascular risk assessment: prediction scores and risk factors

The cardiovascular risk assessment was performed using scores jointly developed by the ACC/AHA (10).

The ACC/AHA algorithm includes the variables sex, age, race, total cholesterol (TC), high-density lipoprotein cholesterol (HDL-c), and systolic blood pressure (SBP) values, treatment for high blood pressure, diabetes, and current smoking status, and estimates the 10-year risk of first atherosclerotic CV disease (ASCVD) event in non-Hispanic African American and non-Hispanic white men and women from 40 to 79 years of age.

Information on race/skin color was obtained by self-classification according to categories adopted by the Brazilian Institute of Geography and Statistics (IBGE) census, those self-declared as black were classified as “African Americans”, and racial/ethnic categories as white, mixed-race, Asians, and indigenous were grouped as “white or others”.

Heart Risk Calculator (HRC, accessed March 2019) was used and enabled risk estimation: ≥7.5% was categorized as elevated 10-year risk of first ASCVD event and <7.5% as low risk, as adopted by the “2013 ACC/AHA Guideline on the Treatment of Blood Cholesterol to Reduce Atherosclerotic Cardiovascular Risk in Adults” (16).

### Blood collection

Blood samples were collected after 12-h overnight fast for determination of total cholesterol (190 mg/dL, as cutoff value) and HDL-c (40 mg/dlL, as cutoff value) (17). IL-6 and IL-10 were evaluated using sandwich ELISA, according to the manufacturer's instruction (BD Biosciences, USA). A standard curve with known concentrations of recombinant cytokines was used to calculate the concentration of each cytokine. The coefficient of variation of cytokines was: IL-6: 2.1% and IL-10: 5.4%. Immunoturbidimetric latex assay using an automatic analyzer (A25 BioSystems, Spain) was used to measure hsCRP. The coefficient of variation for CRP was 1.4%. Plasma leptin (Cat. #ZHL-80SK) and adiponectin (Cat. #EZHADP-61K) levels were determined using a Millipore analysis kit (USA).

### Blood pressure

Blood pressure was measured (OMRON HEM-7113, Japan) three times and the mean value of SBP was determined; <120 and ≥120 mmHg were considered normal and elevated, respectively (18).

### Body composition

Body composition was evaluated from the total body scan by dual energy X-ray absorptiometry (DXA) (Lunar iDXA with software enCore 2008 v12.20, GE Healthcare, USA). Abdominal subcutaneous tissue and VAT were estimated within the android region using the software CoreScan (19). The same operator performed all the scans.

### Statistical analyses

Statistical analyses were performed with the Statistical Package for Social Science (v17.0.1; SPSS Inc., USA) software 19.0. Shapiro-Wilk test was used to test for the homogeneity of the data. All statistical analyses were performed separately according to gender. The participants were stratified according to quartiles (P) of the VAT/SAT ratio (P5, P25, P50, P75) according to sex. Descriptive tables present the results as the median (minimum and maximum) of continuous variables. Kruskal-Wallis test was used to compare more than three groups. Mann-Whitney test was performed to compare the differences between sexes. Spearman's rank correlation (r) was used to observe correlation among the variables. P<0.05 was considered statistically significant.

## Results

Overall, 309 individuals [155 men and 154 women (48.7% of them in menopause)] were included in this study. The median age was 49 years for both sexes. According to median values, males had higher body mass than females (79.9 and 70.7 kg, respectively; P<0.001) with no differences between sexes regarding BMI. Compared to females, males had lower plasma leptin levels (2.4 *vs* 7.6 ng/mL; P<0.001), adiponectin (31.4 *vs* 46.5 ng/mL; P<0.001), CRP (0.14 *vs* 0.24 mg/dL; P<0.001), and SAT (953 *vs* 1551 g; P<0.001), but higher glucose levels (91 *vs* 84 mg/dL; P<0.001), VAT (1436 *vs* 852.5 g; P<0.001), and VAT/SAT ratio (1.3 *vs* 0.5; P<0.001). No differences between sexes were observed for serum IL-10 levels. Also, males had a higher calculated CV risk-score than females (3.9 *vs* 1.2%; P<0.001).

Continuous variables were stratified into quartiles (P5: 0.34 and 0.10; P25: 0.92 and 0.34; P50: 1.33 and 0.52; P75: 1.93 and 0.72 for males and females, respectively) of the VAT/SAT ratio (Table 1). Among males, plasma leptin levels were higher at P75 and P50 than at P5 (2.8 and 2.9 *vs* 1.2 ng/mL, respectively). In addition, higher levels of CRP were detected in males from P75 and P50 (both 0.2 mg/dL) compared to P5 (0.08 mg/dL). Moreover, a higher calculated CV risk-score was observed in males at P75 (7.1%) compared to P5 (2.45%) and P25 (3.3%). Higher plasma adiponectin levels were observed in females at P25 compared to P75 (54.3 *vs* 36 ng/mL, respectively; P<0.05). Lower levels of plasma CRP were observed in females at P5 (0.1 mg/dL) compared to P50 (0.3 mg/dL) and P75 (0.4 mg/dL) (P<0.05). A higher calculated CV risk-score was observed among females at P75 (2.0%) compared to P5 (0.7%) and P25 (1.0%), and P50 (2.1%) compared to P5 (0.7%). In all quartiles of the VAT/SAT ratio, females had higher leptin levels than males, while adiponectin was higher at P5 and P25 in females compared to males (56.1 *vs* 40.3 ng/mL; P<0.05 and 54.3 *vs* 29.4 ng/mL; P<0.05, respectively). The calculated CV risk-scores were higher in males than females in all quartiles of the VAT/SAT ratio (Table 1).

**Table 1:** Inflammatory markers according to quartiles of the VAT/SAT ratio.

	VAT/SAT ratio							
	Male (n=155)				Female (n=154)			
	P5 (n=39)	P25 (n=43)	P50 (n=37)	P75 (n=36)	P5 (n=36)	P25 (n=42)	P50 (n=37)	P75 (n=39)
Leptin (ng/mL)	1.2^a^ (0.2-15.9)	2.3^ab^ (0.2-17.6)	2.9^b^ (0.5-25.7)	2.8^b^ (0.4-14.7)	6.7* (1.3-28)	6.4* (0.6-19.6)	8.1* (2.9-25.5)	9.0* (0.4-28.5)
Adiponectin (ng/mL)	40.3 (0.6-120)	29.4 (3.8-160.5)	31.2 (1.7-137.5)	25.4 (4-123)	56.1^ab^* (16-154.6)	54.3^a^* (16-144)	42.2^ab^ (8.8-168.2)	36.0^b^ (0.5-224.6)
CRP (mg/dL)	0.08^a^ (0.01-0.14)	0.13^ab^ (0.01-1.14)	0.20^b^ (0.01-1.6)	0.20^b^ (0.01-1.5)	0.1^a^ (0.01-1.3)	0.2^ab^* (0.03-2.9)	0.3^b^ (0.03-3)	0.4^b^ (0.01-3.4)
IL-6 (pg/mL)	0.8 (0-19.7)	0.6 (0-12.1)	0.6 (0-13.4)	0.7 (0-17.4)	0.7 (0-14.2)	0.6 (0-47.9)	0.7 (0-9.4)	0.9 (0-13.7)
IL-10 (pg/mL)	0 (0-30)	0.4 (0-275)	0 (0-368)	0.09 (0-243)	0 (0-18.7)	0 (0-124)	0 (0-13)	0 (0-82.8)
Calculated CV risk-score (%)	2.4^a^ (0.6-9.9)	3.3^a^ (0.7-26.9)	4.0^ab^ (1.1-13.3)	7.1^b^ (1.1-32.4)	0.7^a^* (0.2-4.8)	1.0^ac^* (0.2-7.6)	2.0^ab^* (0.2-13.4)	2.0^b^* (0.3-14.9)

Data are reported as median (minimum-maximum). VAT: visceral adipose tissue; SAT: subcutaneous adipose tissue; CRP: C-reactive protein; CV: cardiovascular. Different letters in the same row indicate statistical difference (P<0.05, Kruskal-Wallis test). *P<0.05 for quartile between sexes (Mann-Whitney test).

In males, a positive correlation between leptin and VAT and SAT (r=0.67 and r=0.51, respectively; P<0.001 for both) was observed, whereas a weaker but also positive correlation was observed in females (r=0.29 and r=0.30 respectively; P<0.001 for both). Plasma leptin concentration was positively correlated with the VAT/SAT ratio (r=0.26; P=0.001 and r=0.18; P=0.022) and with CRP (r=0.35; P<0.001 and r=0.23; P=0.005), for males and females, respectively. In contrast, adiponectin levels were inversely correlated with VAT in both sexes (males, r=-0.24; P=0.004; females, r=-0.21; P=0.008) and with VAT/SAT ratio only in females (r=-0.26; P=0.001). Interestingly, adiponectin positively correlated with IL-10 in males (r=0.17; P=0.032) and females (r=0.22; P=0.006). On the other hand, IL-10 did not correlate with VAT, SAT, or VAT/SAT ratio in either sex (data not shown). Calculated CV risk-score was positively correlated with VAT and VAT/SAT ratio in males (r=0.29; P<0.001 and r=0.41; P<0.001, respectively) and females (r=0.42; P<0.001 and r=0.41; P<0.001, respectively). In males, the calculated CV risk-score was also positively correlated with CRP (r=0.18; P=0.027) but inversely with IL-10 (r=-0.19; P=0.020) (Table 2). No statistically significant correlation between SAT and adiponectin or calculated CV risk-score was observed in males and females (Table 2).

**Table 2:** Significant Spearman correlation coefficients (r) among VAT, SAT, VAT/SAT ratio, and inflammatory markers, and calculated CV risk-score according to gender (male, n=155; female, n=154).

	Adipose tissue						Inflammatory markers			
	VAT		SAT		VAT/SAT ratio		IL-10		CRP	
	r	P	r	P	r	P	r	P	r	P
Male										
Leptin	0.67	<0.001	0.51	<0.001	0.26	0.001			0.35	<0.001
Adiponectin	-0.24	0.004	-0.13	0.120	-0.16	0.056	0.17	0.032		
CRP	0.55	<0.001	0.23	0.003	0.35	<0.001				
CV risk-score	0.29	<0.001	-0.15	0.063	0.41	<0.001	-0.19	0.020	0.18	0.027
Female										
Leptin	0.29	<0.001	0.30	<0.001	0.18	0.022			0.23	0.005
Adiponectin	-0.21	0.008	-0.14	0.868	-0.26	0.001	0.22	0.006		
CRP	0.39	<0.001	0.38	<0.001	0.22	0.007				
CV risk-score	0.42	<0.001	0.11	0.174	0.41	<0.001				

VAT: visceral adipose tissue; SAT: subcutaneous adipose tissue; CRP: C-reactive protein; CV: calculated cardiovascular

risk-score; IL: interleukin.

## Discussion

Adipose tissue secretes several bioactive molecules, including adipokines. An imbalance in adipokines production is associated with obesity complications such as type II diabetes and CV risk-score (20). It is well known that obesity is an independent risk factor for CV disease (21,22) and that VAT is more strongly associated with CV disease and inflammation (4). The present study observed an association among the VAT/SAT ratio, inflammatory markers such as leptin and CRP, and calculated CV risk.

Adipose tissue distribution varies by sex (23,24); women generally have a higher fat mass proportion than men with the same BMI. Depending on this distribution, women present higher plasma leptin and adiponectin than men (25,26). In the premenopausal period, women have less VAT than gluteofemoral fat tissue, which leads to differences in adipokine production (27,28).

It has been shown that the VAT/SAT ratio correlates with CV risk-score (29,30). In the present study, it was observed that plasma leptin levels positively correlated with the VAT/SAT ratio in both men and women, whereas adiponectin inversely correlated with the VAT/SAT ratio, especially in women.

Leptin is a pro-inflammatory adipokine involved in increased CV risk-score whereas adiponectin has protective effects on the cardiovascular system (31,32), possibly due to the induction of IL-10 production (33,34), an anti-inflammatory cytokine produced by different cells such as T helper cells, B cells and macrophages. IL-10 is inversely correlated with BMI and multiple metabolic risk factors (35,36). As expected, in the present study, a positive correlation between IL-10 and plasma adiponectin levels in both sexes, and a negative correlation with calculated CV risk-score in males, who presented a higher calculated CV risk-score than females, were observed.

Males seemed to have higher inflammatory markers than females since they had a higher VAT/SAT ratio and higher calculated CV risk. Conversely, no difference between men and women regarding levels of the pro-inflammatory cytokine IL-6 or the anti-inflammatory IL-10 was found. Possibly, the inverse correlation between IL-10 and calculated CV risk-score observed only among men is a metabolic attempt to limit inflammation, protecting them from CV disease (35,36). It has been shown that CRP is related to different adiposity indices such as total body fat and VAT (37,38). The present study shows higher CRP levels in both men and women with a higher VAT/SAT ratio. Additionally, a positive correlation between CRP and calculated CV risk-score was found, in agreement with several previous studies suggesting that molecules associated with low-grade inflammation, such as CRP, are predictors of CV diseases (39).

In the present study, men presented a higher VAT/SAT ratio and calculated CV risk-score than women. Since VAT is biologically more active, contributing to low-grade inflammation, it could be concluded that fat distribution could be a better indicator of CV disease than overall adiposity. Notably, fat distribution and the VAT/SAT ratio are influenced by sex. Therefore, our results strengthened the relevance of the association of VAT/SAT ratio with cardiovascular risk.
